# Multi-Task Foreground-Aware Network with Depth Completion for Enhanced RGB-D Fusion Object Detection Based on Transformer

**DOI:** 10.3390/s24072374

**Published:** 2024-04-08

**Authors:** Jiasheng Pan, Songyi Zhong, Tao Yue, Yankun Yin, Yanhao Tang

**Affiliations:** 1 School of Computer Engineering and Science, Shanghai University, No. 99 Shangda Road, Shanghai 200444, China; pjs199999@shu.edu.cn; 2 School of Mechatronic Engineering and Automation, Shanghai University, No. 99 Shangda Road, Shanghai 200444, China; tao_yue@shu.edu.cn; 3 School of Artificial Intelligence, Shanghai University, No. 99 Shangda Road, Shanghai 200444, China; yankun_yin@shu.edu.cn (Y.Y.); yanhao@shu.edu.cn (Y.T.)

**Keywords:** point cloud data, YOLO, Transformer, multi-source feature fusion, depth completion

## Abstract

Fusing multiple sensor perceptions, specifically LiDAR and camera, is a prevalent method for target recognition in autonomous driving systems. Traditional object detection algorithms are limited by the sparse nature of LiDAR point clouds, resulting in poor fusion performance, especially for detecting small and distant targets. In this paper, a multi-task parallel neural network based on the Transformer is constructed to simultaneously perform depth completion and object detection. The loss functions are redesigned to reduce environmental noise in depth completion, and a new fusion module is designed to enhance the network’s perception of the foreground and background. The network leverages the correlation between RGB pixels for depth completion, completing the LiDAR point cloud and addressing the mismatch between sparse LiDAR features and dense pixel features. Subsequently, we extract depth map features and effectively fuse them with RGB features, fully utilizing the depth feature differences between foreground and background to enhance object detection performance, especially for challenging targets. Compared to the baseline network, improvements of 4.78%, 8.93%, and 15.54% are achieved in the difficult indicators for cars, pedestrians, and cyclists, respectively. Experimental results also demonstrate that the network achieves a speed of 38 fps, validating the efficiency and feasibility of the proposed method.

## 1. Introduction

The perception of surrounding objects is crucial for autonomous driving [1]. However, the dynamic characteristics of objects are influenced by environmental factors like lighting, fog, rain, wind, and reflections. Challenges in distinguishing foreground from background arise due to factors like rainwater or oil obstructions, decreased visibility due to fog, blurring from motion, and color distortions. These challenges significantly complicate the task for recognition algorithms that depend on RGB images. The advent of depth sensors has rendered depth images more attainable, providing depth disparity information between foreground and background to enhance object detection. Depth images play a pivotal role in various practical applications, including stereo matching [2], image understanding [3], co-saliency detection [4], action recognition [5], video detection and segmentation [6,7,8,9], semantic segmentation [10,11], medical image segmentation [12,13,14], object tracking [15,16], camouflage object detection [17], and image retrieval [18].

Nevertheless, most of the depth images utilized in these methods originate from depth cameras. In outdoor settings, depth cameras are susceptible to lighting conditions, with a limited detection range of merely 20 m. Depth images captured beyond this range exhibit substantial errors and noticeable noise [19,20,21,22]. In contrast, LIDAR sensors are less affected by lighting conditions, and multi-line LIDAR sensors can often detect distances exceeding 100 m. Consequently, numerous studies opt for LIDAR to produce depth images and integrate them with RGB cameras.

Currently, a variety of target recognition algorithms incorporate both LIDAR and RGB data. For instance, EPNet [23] and MV3D [24] both project sparse LIDAR data onto the front view. In contrast, SPLATNet [25] maps pixels onto sparse point clouds and derives classification probabilities for each point using 1×1 convolutional kernels, ultimately yielding 3D detection results. However, the methods mentioned above have not adequately addressed the sparsity issue in LIDAR data compared to RGB pixels, leaving significant room for improvement.

These depth maps are then projected into the 3D point cloud coordinate space, and neural networks are employed for object detection. Noteworthy examples of such studies include Pseudo LiDAR [26] and Pseudo LiDAR++ [27]. While these approaches heavily rely on 3D recognition networks, they tend to overlook the potential of mature and stable 2D object recognition algorithms. Other researchers aim to establish a connection between point clouds and 2D semantics by matching semantic segmentation information from images with depth maps. Illustrative examples of such research include Complex-YOLO [28] and MVP [29], guiding 3D networks in object recognition. However, these methods do not fully exploit the information from depth maps to enhance image recognition. Moreover, some studies utilize depth maps generated under image guidance to enhance RGB-based object recognition. Ophoff [30], Chu [31], and Liang [32] leverage dense depth maps for feature extraction, subsequently fusing them with RGB features through concatenation and addition operations across multi-scale feature maps. Nevertheless, these approaches occasionally disregard the disparities between depth map features and RGB features. Shen [33] leverages RGB guidance for LIDAR depth completion, inputting concatenated sparse depth maps, dense depth maps, and RGB images into the YOLO backbone network without further fusion in the FPN (Feature Pyramid Network) phase, resulting in the underutilization of depth map information.

The fusion strategies employed in the above-mentioned methods, which rely on dense depth maps and RGB fusion, often disregard the feature disparities between depth maps and RGB images. Furthermore, these methods concatenate the depth completion network with the recognition network, resulting in reduced recognition efficiency. Depth maps are essentially single-channel images that encapsulate depth values, rich in distance-related information. Notably, they exhibit substantial differences in distance and feature boundaries between foreground and background when compared to RGB images. This inherent property makes them less susceptible to issues arising from texture and color interference. Hence, they are distinctly advantageous in segregating the foreground from the background and accentuating target information. In light of these advantages, we leverage dense depth maps to dynamically generate weight values for each pixel at various scales, and subsequently apply these weights to the RGB feature maps. This strategy steers the detection module towards a more focused assessment of color and texture features within the target area, thus reducing false positives and enhancing recognition rates.

Our contribution can be summarized as follows:

1. We introduce a real-time object recognition and depth completion approach using a single Transformer backbone network, allowing the simultaneous extraction of RGB and sparse LIDAR features. Compared to the 32 ms required when serializing the depth completion network and object detection network, our integrated network, inclusive of object detection functionality, totals an inference time of 26 ms.
2. During the Feature Pyramid Network (FPN) stage, we use a weight matrix based on dense depth map features to enhance the detection of small and challenging objects. This approach yields significant improvements in detection accuracy for vehicles, bicycles, and pedestrians within the Kitti dataset, showing relative enhancements of 4.78%, 8.93%, and 15.54%, respectively, over the baseline.
3. We generate masks for regions with target recognition labels, calculate depth completion loss separately, and reduce the weight of depth completion loss in environmental areas to mitigate noise impact on the neural network.

## 2. Related Work

### 2.1. General 2D Image Object Detection

Deep learning-based detection algorithms are typically divided into two main categories: two-stage and one-stage detectors. The well-known RCNN series, including RCNN [34], Fast RCNN [35], and Faster RCNN [36], are two-stage algorithms known for their superior accuracy over many other detection methods. Nonetheless, they are computationally intensive, resulting in longer processing times. On the other hand, one-stage detectors like SSD (Single Shot MultiBox Detector) [37] and YOLO (You Only Look Once) were developed to strike a balance between accuracy and efficiency. Particularly, YOLO is renowned for its effectiveness in balancing these two aspects.

RCNN utilizes a strategy based on region proposals [38], where each proposal is normalized in scale before being classified by a ConvNet [39]. Advanced detectors such as Fast RCNN and Faster RCNN promote the usage of features calculated at a singular scale, optimizing the balance between accuracy and processing duration. However, these methods are not fast enough for embedded board applications due to their high memory demands and complex network architectures, making real-time performance difficult to achieve [40,41].

Focusing on efficiency, one-stage object detection methods have gained significant interest. The SSD technique, introduced by Liu et al. [42], assigns different scale anchors across multiple ConvNet layers, with each layer tasked to predict objects of a certain scale. To further enhance SSD, Fu et al. [43] developed the Deconvolutional Single Shot Detector (DSSD), integrating Residual-101 [44] with SSD and adding deconvolutional layers. This provides a broader scale context for detection, thereby improving accuracy. Another innovation by Li et al. [45], the Feature Fusion Single Shot Detector (FSSD), augments SSD with a novel, lightweight feature fusion module. This module connects multi-layer features from various scales to create a new feature pyramid using downsampling blocks, which is then utilized for final detection predictions.

YOLO performs object category and position predictions through a singular forward convolutional network, achieving impressive frame rates of up to 45 fps. Its successor, YOLOv2 [46], made several enhancements, including the adoption of high-resolution layers, batch normalization in each convolutional layer, and the use of convolutional layers for bounding box predictions. YOLOv3 [47] further improved the framework by switching to the darknet-53 backbone network and incorporating multi-scale feature utilization for detection. YOLOv5 [48] introduced new elements such as the Focus module, SPP module, and a feature pyramid structure. These enhancements allow for the fusion of multi-scale features at different stages of detection, thus boosting accuracy and stability.

### 2.2. Multi-Sensor Fused Object Detection

Intelligent vehicles have increasingly adopted the integration of camera and LiDAR technologies for detecting objects. The approaches to this fusion have evolved over time. The Navlab team utilized a combination of multiple cameras and laser scanners to identify moving objects, as outlined in Aufrère et al.’s work [49]. They employed image-based edge detection and used laser scanners for edge localization, classifying objects based on their motion trajectories. Monteiro et al. [50] implemented a single laser scan and a camera for object detection in semi-structured outdoor environments tailored to intelligent vehicles. Their approach involved using the laser for rapid detection to create regions of interest (ROIs), followed by the application of two distinct classifiers to these ROIs for obtaining individual results. In similar vein, Premebida et al. [51] designed a perception system, optimized for pedestrian detection, that employed two distinct fusion architectures, both enhancing detection capabilities.

With the increasing integration of LiDAR technology in autonomous driving systems, many studies have combined camera and LiDAR data for 3D object detection via Deep Convolutional Neural Networks (DCNNs) [52,53]. The PC-CNN framework [52] offers a method to extend 2D object detection to 3D by utilizing images to generate 2D detection results, which are then used to narrow down the search area in the point cloud. However, this framework does not exploit the point cloud data to enhance 2D detection performance, representing a promising area for further development. F-PointNet [53] follows a similar procedure, generating 3D frustums from the point cloud based on 2D detection results, and then conducting 3D instance segmentation based on 3D box estimates. However, in this approach, image and point cloud data are processed using separate branches without in-depth fusion, missing an opportunity to improve 2D detection capabilities. F-PointNet [53] introduced a comparable workflow, producing a 3D frustum from the point cloud based on 2D detection outcomes, followed by 3D instance segmentation in line with 3D box estimation. Yet, this method did not deeply fuse image and point cloud data for feature extraction, thus missing an opportunity to enhance 2D detection capabilities.

In this paper, a critical issue addressed is how to directly extract features from the integrated data space of images and point clouds, instead of merely conducting a fusion process post individual feature extraction. Additionally, inspired by the works of Ochs [54] and Klingner [55], we recognized the parallelizability of depth completion and object detection tasks. Consequently, we have implemented this objective through Transformer, resulting in relatively favorable outcomes.

## 3. Methodology

**Figure 1:**
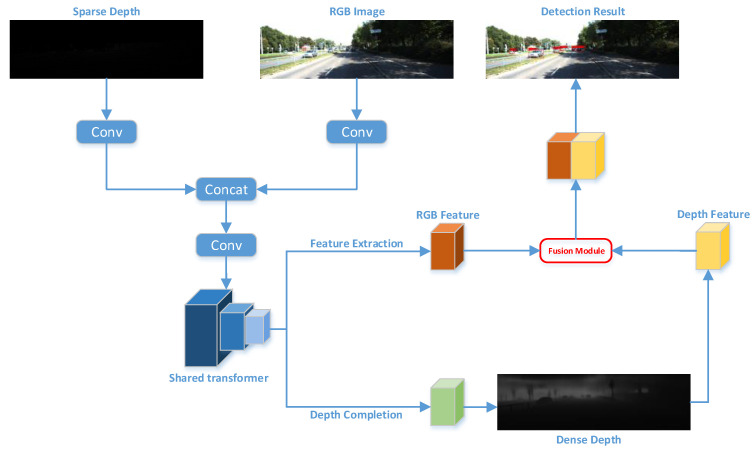
Network architecture diagram.

Existing object detection networks typically use the results of the depth completion network as input to the object detection network and perform fusion in a sequential manner. However, during the fusion stage, these networks often rely solely on the Concat method, which not only affects real-time performance but also does not yield significant fusion improvements. As illustrated in Figure 1, to maintain real-time processing, our approach conducts object detection and depth completion concurrently within the same neural network backbone. Furthermore, in the subsequent Feature Pyramid Network (FPN) stage, we leverage the recovered dense depth map features for further fusion, thereby enhancing fusion effectiveness.

Compared to YOLOv5, our model substitutes the backbone with a Swin-Transformer [56] and integrates a preprocessing network prior to the Swin-Transformer, aiming for the more effective extraction of features from sparse depth maps and RGB images independently. Additionally, we added a neural conditional random field-based depth completion branch downstream to reconstruct dense depth maps. Furthermore, we incorporated a fusion module to extract dense depth map features and fused them with RGB and sparse LIDAR features.

### 3.1. Data Augmentation

The commonly used data augmentation methods in YOLO include Mosaic, CutMix, and MixUp. The Mosaic method involves processing four images using basic data augmentation techniques such as cropping and scaling, and then randomly combining them to generate a new image. The CutMix method randomly selects two images from the dataset, crops one of them, overlays it onto the other image, and then inputs the newly generated image into the network for training. However, this can result in discontinuities in depth maps. The MixUp method combines two images without cropping, applies different opacities to overlap them, and then inputs them into the network, causing a single pixel to have two depth values.

Considering the unique characteristics of depth completion tasks, depth values within a single image often exhibit continuity, and the same pixel location cannot have two depth values simultaneously. Therefore, we have excluded the CutMix and MixUp algorithms and ultimately opted for the Mosaic method. In our approach, we scale and combine four depth maps with RGB images proportionally and then input them into the network for training.

### 3.2. Preprocessing Module

In the backbone network stage, we used Swin-Transformer-tiny as the base network, which is superior to CSPN-Darknet in extracting global features, which is crucial for both depth completion and object detection tasks. Since RGB images have dense pixels and a higher number of channels, while sparse depth maps have sparse effective pixels and are single-channel, there is a significant difference in the amount of information carried by the two. Sparse depth maps, due to their sparse effective depth, require a larger receptive field than RGB images to extract effective features. Directly inputting them into the network would result in the network’s inability to allocate the number of feature maps accurately for different sensor data.

Hence, we refrained from directly feeding them into the network; instead, we applied three 3×3 convolutional layers for preprocessing. This approach allowed us to intervene manually in the number of feature map channels occupied by each sensor data and also increased the receptive field for the sparse depth map. This strategy effectively prevents the premature loss of substantial RGB or depth feature information during the integration of feature layers. The formula is as follows:

(1) $$F_{RGB}=Conv3\left(f_{RGB},{Outchannel}_{RGB}\right),$$

(2) $$F_{Depth}=Conv3\left(f_{Depth},{Outchannel}_{Depth}\right),$$

(3) $$F_{Final}=Conv3\left(Concat\left(F_{RGB},F_{Depth}\right),{Outchannel}_{Union}\right),$$

where Conv3 represents a 3×3 convolution, $f_{RGB}$ denotes the initial input three-channel RGB image, $f_{Depth}$ represents the initial input single-channel depth map, Concat indicates the stacking of feature maps along dimensions, and ${Outchannel}_{RGB}$, ${Outchannel}_{Depth}$, ${Outchannel}_{Union}$ are set to 48, 16, and 64, respectively, representing the number of channels in the output after convolution.

### 3.3. Depth Completion

**Figure 2:**
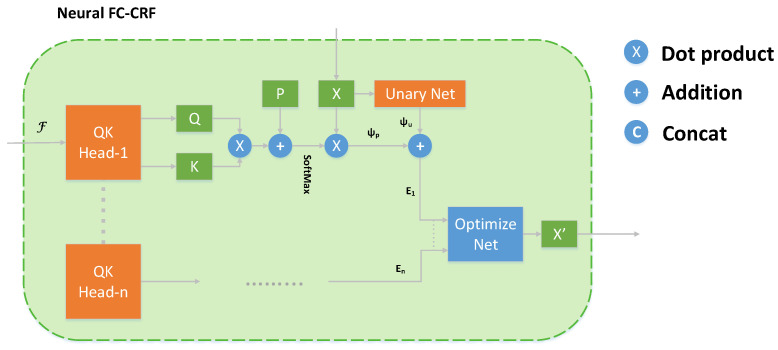
In the Neural FC-CRF, we begin with the initial prediction *X* based on image and sparse depth features *F*. Subsequently, at each level, the network constructs a multi-head attention mechanism from *X* and *F* to optimize for an improved prediction $X^{\prime }$.

For the features extracted at different scales by the backbone network, we feed them separately into the FPN (Feature Pyramid Network) and the depth completion network. In the FPN section, we drew inspiration from the design philosophy of YOLOv5. As for the depth completion section, we employed a neural conditional random field [57], as illustrated in Figure 2.

In our research, Conditional Random Fields (CRF) are utilized to enhance the accuracy of depth estimation. The estimation of depth associated with a given pixel is influenced by surrounding pixels within a broadened scope across the image. In the context of graphical models, the energy function for a fully connected CRF is generally defined as follows:

(4) $$E\left(x\right)=\sum_{i}\;\psi_{u}\left(x_{i}\right)+\sum_{ij}\;\psi_{p}\left(x_{i},x_{j}\right),\;$$

in our model, the variable $x_{i}$ is assigned to represent the output prediction for the node labeled *i*. Meanwhile, *j* refers to all remaining nodes within the same graph. For each node, a unary potential, denoted as $\psi_{u}$, is computed, drawing on the information from feature maps. In addition, a pairwise potential, $\psi_{p}$, is established, connecting a given node not just with its immediate neighbors but with every other node in the graph. The unary potential originates from the feature maps processed by the network. On the other hand, the pairwise potential is a composite measure, incorporating the values from both the focused node and all others, factoring in a weight based on the combined attributes of color, depth, and spatial location for each pair of nodes. The mathematical expressions defining these potentials are structured as follows:

(5) $$\psi_{u}\left(x_{i}\right)=N_{u}\left(F,x_{i}\right),$$

(6) $$\psi_{p}\left(x_{i},x_{j}\right)=w\left(p_{i},p_{j},F_{i},F_{j}\right)∥x_{i}-x_{j}∥,$$

in this context, *F* represents the feature map, with *N* denoting the parameters of a unary network. The variable $p_{i}$ indicates the spatial location of node *i*, and *w* is identified as the weighting function.

Furthermore, it is essential that the weights assigned to potential functions differ when considering a node in relation to various other nodes. The redefined potential functions, incorporating these adjustments, are presented as follows:

(7) $$\psi_{x_{i}}=\alpha \left(p_{i},p_{j},F_{i},F_{j}\right)x_{i}+\sum_{j\neq i}\;\beta \left(p_{i},p_{j},F_{i},F_{j}\right)x_{j},\;$$

in this formulation, α and β function as weighting factors, determined through network computation.

Our method is influenced by recent advancements in Swin-Transformer technology. For each patch within a given window, we derive query vectors *q* and key vectors *k* from their respective feature maps. These vectors, aggregated from all patches, are then formulated into matrices represented as *q* and *k*. Following this, we calculate the dot product of these matrices to ascertain potential weights that define pairwise relationships. The final pairwise potentials are obtained by scaling the predicted values *X* with these deduced weights.

**Figure 3:**
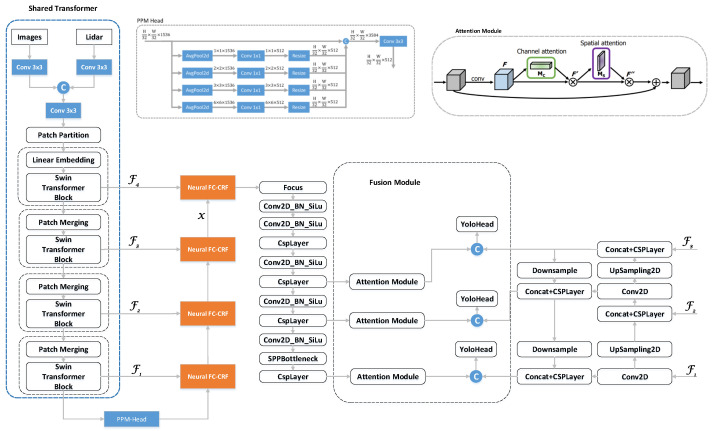
The final densely completed feature map is re-extracted for feature enhancement. Channel and spatial attention mechanisms are applied at different scales to weight the regions of interest and are concatenated with the features extracted by YOLO before being sent to the head for detection. $F_{1}$, $F_{2}$, $F_{3}$ represent shared feature maps, each being input into the depth completion and FPN stages.

To incorporate spatial context, relative position embeddings, denoted as *P*, have been integrated into our model. Consequently, the calculation of the previously mentioned formula is executed as follows:

(8) $$\psi_{p_{i}}\;=SoftMax\left(q\cdot K^{T}+P\right)\cdot X,$$

(9) $$\sum_{i}\;\psi_{p_{i}}\;=SoftMax\left(Q\cdot K^{T}+P\right)\cdot X,$$

where · represents dot product, the output of SoftMax yields weights α and β, determining the weights of information with position encoding *P*. The final module structure is shown in Figure 3.

### 3.4. Fusion Module

During the Feature Pyramid Network (FPN) phase, preceding the final detection stage, features processed through depth completion undergo convolution and downsampling. These enhanced features are then integrated with the semi-dense depth feature map. The fusion of RGB and depth features presents two main challenges: firstly, a compatibility issue due to the inherent differences between the modalities, and secondly, the presence of redundancy and noise in low-quality depth features. Inspired by [58], we have developed a depth-enhancement module. This module is tailored to augment the synergy of multi-modal features and to distill valuable information from the depth feature maps, as depicted in Figure 3.

Specifically, we denote $f_{i}^{rgb},\;f_{i}^{d}$ as the feature maps from the *i*-th side output layer of the RGB and depth branches, respectively. Each fusion module is added before introducing features from the depth branch into each side output feature map, aiming to improve the compatibility of these depth feature maps. This lateral output procedure not only enhances the prominence of depth feature maps but also conserves information across multiple levels and varying scales. The methodology for integrating features in both scenarios is characterized as follows:

(10) $$f_{DEM}\left(f_{i}^{d}\right)=S_{att}\left(C_{att}\left(f_{i}^{d}\right)\right),$$

(11) $$f_{i}^{cm}=Concat\left(f_{i}^{rgb},f_{DEM}\right),$$

where $f_{i}^{cm}$ represents the feature maps from the *i*-th layer of multimodal fusion, and the DEM module encompasses both sequential channel attention mechanism and spatial attention mechanism, $S_{att}$ represents the spatial attention module, and $C_{att}$ represents the channel attention module.

### 3.5. Loss Function Design

In the phase of depth completion, our approach incorporated the use of Scale-Invariant Logarithmic (SILog) loss, as detailed in [59], for overseeing the training process. With access to the ground truth depth map, our first step was to compute the logarithmic deviation between the forecasted depth map and the ground truth depth measurement. Subsequently, we computed the scale-invariant loss to ensure the effective supervision of the model training even in situations with varying scales:

(12) $$\Delta d_{i}=log{\hat{d}}_{i}-logd_{i}^{*},$$

(13) $$L=\alpha \sqrt{\frac{1}{K}\sum_{i}\;\Delta d_{i}^{2}-\frac{\lambda }{K^{2}}{\left(\sum_{i}\;\Delta d_{i}\right)}^{2}},$$

where $d_{i}^{*}$ represents the true depth value, $d_{i}$ is the predicted depth value for pixel *i*. λ is the variance minimization factor, set to 0.85, and α is a scale constant, set to 10.

**Figure 4:**
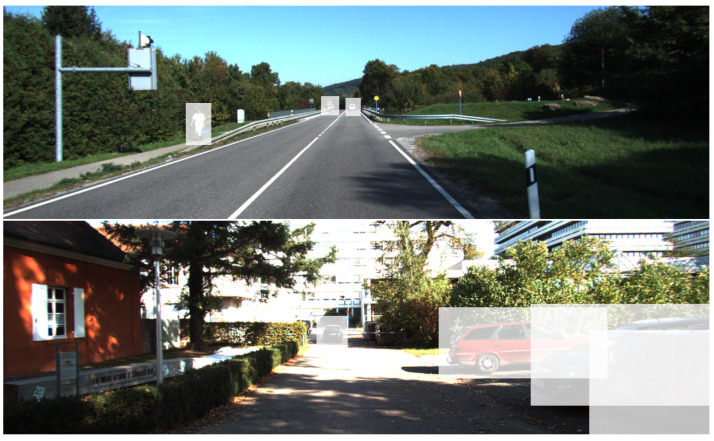
Previous depth completion loss calculations required assessing the valid depth areas across the entire image. In this paper, we leverage target boxes derived from object detection to generate masks (represented by the semi-transparent areas in the image) for the isolated computation of depth completion loss in regions containing targets.

In most scenes, areas containing branches, grass, and shrubs are common, and these areas often contain a significant amount of noise, occupying a large portion of the image. At the same time, depth ground truth labels are also unable to accurately represent them. Therefore, we have employed label data from object detection to generate masks (as shown in the semi-transparent rectangular areas in Figure 4) to ignore most of the background areas. By calculating the depth completion loss for both the masked areas and the entire image separately, we can effectively reduce the loss weight of the background areas, allowing the network to fit better. The final depth completion loss is designed as follows:

(14) $$L_{mask}=\alpha \sqrt{\frac{1}{K}\sum_{i}\;\Delta d_{i-mask}^{2}-\frac{\lambda }{K^{2}}{\left(\sum_{i}\;\Delta d_{i-mask}\right)}^{2}},$$

(15) $$L_{depth}=\;L+{0.5\times L}_{mask},$$

where $\Delta d_{i-mask}$ represents the logarithmic difference between the predicted depth map and the actual depth in the masked region.

The object detection loss consists of anchor localization loss computed using Generalized Intersection over Union (GIOU), cross-entropy loss for classification, and confidence loss. Finally, our loss function is designed as follows:

(16) $$L_{total}=\;\beta \left(L_{BCEcls}+L_{BCEobj}+L_{giou}\right)+L_{depth},$$

where β is a scalar parameter, $L_{BCEcls}$ represents cross-entropy loss for classification, $L_{BCEobj}$ represents cross-entropy loss for confidence loss. To adjust the neural network predominantly by depth completion in the early training stages and by the detection task in the later stages, we set β to 0.1 for the first 100 epochs, and then to 1 and 10 at epochs 100 and 150, respectively.

### 3.6. Dataset and Metric

Given that KITTI provided the necessary dense depth ground truths and object detection labels for our training, we conducted our training and testing on this dataset. The training set for the KITTI object detection task comprises 7481 paired instances. Of these, 3740 pairs are allocated for training purposes, and 3741 for testing. Addressing the skewed distribution of object categories within KITTI, categories such as ‘Car’, ‘Van’, ‘Truck’, and ‘Tram’ were consolidated under the single label ‘Car’, while both ‘Pedestrian’ and ‘Person sitting’ were combined under ‘Pedestrian’. Our analysis primarily focused on the categories of ‘Car’, ‘Pedestrian’, and ’Cyclist’. In the training phase, mosaic augmentation techniques were employed, and random adjustments in translation, orientation, and scale were applied to both LIDAR point clouds and camera images. For concurrent task training, the KITTI depth dataset provided dense depth annotations. The KITTI benchmark uses Average Precision (AP), calculated at 40 distinct points on the Precision–Recall (PR) curve, as its detection metric. The 2D assessment criterion for cars requires an Intersection over Union (IoU) of 0.7, whereas, for other categories, the IoU threshold is set at 0.5. KITTI further categorizes object labels into three groups based on size and degree of occlusion, namely easy, moderate, and hard.

### 3.7. Implementation Details

We detect objects in the RGB images where LIDAR points fall because only these regions can benefit from image feature augmentation. We also crop different-sized images to a uniform size of 352×1216 pixels. Model training was conducted on a single GPU machine with a total batch size of 6. We set the initial learning rate to 0.0001 with a minimum learning rate of 0.000001. Utilizing the Adam optimization algorithm (β1 = 0.9, β2 = 0.999), along with a cosine annealing strategy with $T_{max}$ set to 40 epochs, we trained the model for a total of 250 epochs. The methodology we have developed was executed on a single RTX 3090 graphics card, equipped with 24 GB of memory.

### 3.8. Evaluation Metrics

Evaluation metrics for object detection models based on deep learning include recall, precision, average precision (AP), accuracy, and others. Precision quantifies the ratio of correctly detected objects out of all detected objects. Recall gauges the proportion of correctly predicted positive samples out of all positive samples. Accuracy assesses the ratio of correctly predicted objects out of all objects.

(17) $${Average\;Precision|}_{R}=\frac{1}{\left|R\right|}\sum_{r\in R}p_{interp\;}\left(r\right),$$

where $p_{interp\;}\left(r\right)$ denotes the interpolated average precision at a given recall value *r*, *R* represents the number of interpolation points for the average precision. Object detection accuracy and detection coverage are generally evaluated using the Precision–Recall (PR) curve. Higher precision at a fixed recall indicates the better detection capability of the algorithm. In this context, we set *R* to 40.

## 4. Results

### 4.1. Evaluation Results

We compared our method with state-of-the-art detectors in Table 1 and demonstrated that our method outperforms competitors significantly in both pedestrian and cyclist detection tasks. This is mainly due to the unique characteristics of pedestrian and cyclist contours on the depth map, making them easier to distinguish. In contrast, the depth map of vehicles, such as trucks and trams, has relatively small differences from the walls. In poor lighting conditions, it is easy to misidentify walls as vehicles, which somewhat reduces the recognition rate. In the end, under the challenging difficulty level, we achieved a performance improvement of 4.59% and 11.32% compared to the state of the art.

**Figure 5:**
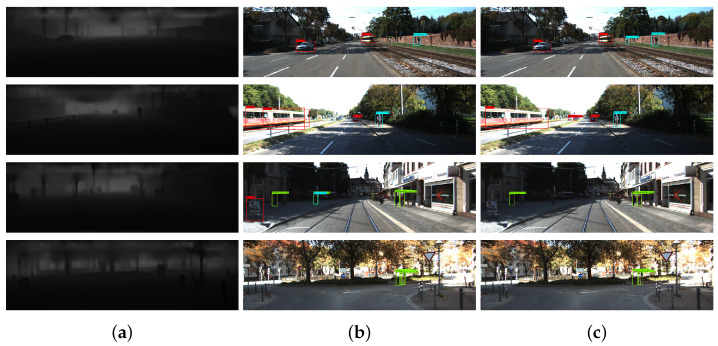
Detection results on the KITTI dataset. (**a**) Generated dense depth map. (**b**) Detection results by concatenating the RGB image with sparse depth map in the detection network. (**c**) Our detection results.

As shown in Figure 5, compared to methods involving concatenating sparse LiDAR data with RGB images, our approach excels in detecting smaller objects and exhibits reduced susceptibility to false positives.

### 4.2. Ablation Study

In this section, our primary emphasis is on the following key aspects:

1. The advantages of employing the preprocessing network in the context of fused object detection.
2. The efficacy of multi-scale fusion during the Feature Pyramid Network (FPN) stage for our fused object detection.
3. The impact on object detection performance due to the design of the loss function.

#### 4.2.1. Impact of the Preprocessing Module

We performed an ablation study using the KITTI dataset, where YOLOV5, augmented with a Swin-Transformer backbone, served as our baseline model. This approach allowed us to evaluate the individual contributions of each component in the system. Our investigation aimed to understand the differences between directly concatenating the LiDAR depth map and RGB image and utilizing a preprocessing convolutional network before feeding them into the Swin-Transformer. As depicted in Table 2, the results show that the preprocessing network leads to an improvement of over 0.5% for detecting hard targets. This improvement can be attributed to several factors. Firstly, the convolutional processing of LiDAR and RGB images within the preprocessing network expands the receptive field, allowing for a better grasp of the data from different sensors. Secondly, it helps control the proportion of feature map layers dedicated to different sensor inputs. Lastly, employing separate convolutional kernels for each sensor data stream makes it more feasible for the network to fit and extract distinctive features from each type of sensor data.

#### 4.2.2. Impact of the Multi-Scale Depth Completion Fusion Module

This module initially employs a neural Conditional Random Field (CRF), which has recently been applied in depth completion, semantic segmentation, and other areas. Its primary function is based on the color and distance correlation between pixels within the image to predict the depth values of pixels with missing depth. After sequentially restoring to the original image dimensions, we use a lightweight feature extraction network to conduct specialized feature extraction on the depth map. Drawing inspiration from the multi-scale design concept in YOLO, we also divide depth features into three scales: small, medium, and large. This allows the model to better adapt to complex scenes, enhancing its perception of objects at different scales and thereby improving the model’s performance across various tasks. We utilize a spatial attention mechanism to calculate the weight of each pixel in space, enabling the model to focus on more important image areas through depth differences while ignoring less significant areas. Concurrently, a channel attention mechanism is used to determine the importance of each channel, with the final combined RGB features then fed into YOLO’s detection head for object detection.

We performed a comparison between the network with and without the multi-scale depth completion fusion module. The results clearly show that the inclusion of features extracted from the dense depth map leads to more precise matching relationships between pixels and LiDAR points. This improvement in matching enhances the detector’s ability to distinguish between foreground and background, leading to notable improvements in detection performance across all levels of difficulty. In particular, the Average Precision (AP) for cars increased by 1.94%, for pedestrians by 5.75%, and for cyclists by 12.71%.

#### 4.2.3. The Combined Impact of the Preprocessing Module and Multi-Scale Fusion Module

Based on the aforementioned observations, we have discovered that the preprocessing network significantly enhances the receptive field of downstream tasks, thereby improving object detection performance. Simultaneously, it endeavors to preserve RGB features while mitigating the dominance of depth-related features within the channels. We integrated the preprocessing network with other modules, and through experimentation, we observed that the preprocessing network, by simultaneously expanding the receptive field for both depth completion and object detection tasks, exhibits a more pronounced impact on networks equipped with the multi-scale depth completion fusion module compared to networks lacking this design module.

#### 4.2.4. Impact of the Loss Function Design

The enhancement of the loss function has led to a more focused depth completion on areas with cars, pedestrians, and cyclists, thereby reducing the impact of environmental noise on depth completion. Consequently, the depth completion of targeted areas has become more refined, which in turn has improved the effectiveness of object detection after the integration of depth features.

Ultimately, in comparison to the baseline, vehicle recognition improved by 4.78%, pedestrian recognition improved by 8.93%, and cyclist recognition improved by 15.54%.

## 5. Discussion

MSF-YOLO [33] simply concatenates the dense depth map with RGB data, without employing advanced fusion techniques. This basic method of fusion fails to exploit the depth information fully, leading to inadequate detection performance. Furthermore, GFD-Retina [60] advances upon this by designing a fusion unit that merges depth features with RGB features at multiple scales, thereby outperforming MSF-YOLO in pedestrian and cyclist detection. However, methods mentioned above did not utilize shared feature extraction between depth completion and object detection, resulting in redundant feature extractions and longer processing times (32 ms). On the other hand, the environmental noise was not considered in depth completion loss functions, resulting in only a 2.4% improvement in prediction accuracy. VPF [61] employs computationally intensive 3D convolutions and complex fusion modules. It achieves an average precision of 78.86% across all classes but requires inference times exceeding 70 ms. Contrarily, our method shares a feature extraction network between depth completion and object detection, merging depth and RGB features before the final detection stage. This strategy boosts the network’s inference speed and reduces computational demands. Compared to the 32 ms needed for serial integration of depth completion and object detection networks, our enhanced framework requires only 26 ms for inference. Moreover, it outperforms VPF by 4.59% and 11.32% for pedestrian and cyclist detection under the hard level, respectively.

Regarding car recognition rates, the proposed methodology does not exceed the current state-of-the-art. This shortfall primarily stems from suboptimal lighting conditions, under which entities like walls resemble trucks in LiDAR scans, exacerbated by insufficient RGB texture data, resulting in erroneous identifications. Additionally, the depth completion module consumes a considerable segment of inference time; thus, accelerating the computational efficiency of the depth completion module is one of the future research directions.

## 6. Conclusions

We have developed a multi-sensor detection framework that capitalizes on depth completion techniques. This framework uniquely incorporates depth data during both the feature extraction phase and the Feature Pyramid Network (FPN) stage, in conjunction with RGB features. Distinct from conventional fusion methods, our model ensures a more accurate alignment of LiDAR point features with RGB features, both quantitatively and spatially. This alignment fosters enhanced learning of representations and a more robust fusion of dense features. Our methodology, rigorously tested on the KITTI benchmark, has consistently led in performance against the baseline across various detection tasks. Specifically, within the categories classified by KITTI as easy, moderate, and hard, our approach has demonstrated substantial improvements over the baseline. For the ‘hard’ difficulty level, it achieved enhancements of 4.78% for cars, 8.93% for pedestrians, and 15.54% for cyclists, illustrating its robustness in the most challenging scenarios. Future developments focus on enhancing 3D object localization by developing a pseudo point cloud correction module and 3D bounding box fusion algorithm, and adapting the network for instance segmentation.

## Figures and Tables

**Table 1:** Comparative assessment of different methods in 3D object detection, evaluated by Average Precision (AP, %) on the KITTI test dataset. * indicates that the data originate from the referenced paper. Bold indicates the highest recognition rate.

Method	Runtime	Input Data	Car (%)			Pedestrian (%)			Cyclist (%)		
			Easy	Mod	Hard	Easy	Mod	Hard	Easy	Mod	Hard
MMF [32]	43 ms	Image + Lidar	91.82	90.17	88.54	N/A	N/A	N/A	N/A	N/A	N/A
MSF-YOLO [33]	32 ms *	Image + Lidar	95.34	91.12	84.55	75.04	59.03	54.65	66.53	48.23	42.61
Faster R-CNN [35]	24 ms	Image	88.97	83.16	72.62	79.97	66.24	61.09	72.40	62.86	54.97
GFD-Retina [60]	376 ms *	Image + Lidar	94.36	88.54	78.74	77.43	60.00	56.01	79.90	60.43	53.62
VPF [61]	72 ms	Image + Lidar	96.06	**95.17**	**92.66**	75.03	65.68	61.95	82.60	74.52	66.04
YOLOX [62]	18 ms	Image	93.15	87.26	84.49	73.80	65.93	57.81	79.49	71.83	59.38
YOLOV7 [63]	15 ms	Image	94.20	88.13	86.34	73.62	65.91	57.13	79.83	74.15	62.05
Ours	26 ms	Image + Lidar	**96.41**	90.01	89.89	**80.84**	**73.67**	**66.54**	**87.55**	**83.19**	**77.36**

**Table 2:** Ablation study of different modules in the network. “P” refers to the preprocessing module, “L” refers to loss function design, and “M” refers to the multi-scale depth completion fusion module. Bold indicates the highest recognition rate.

Method	Car (%)			Pedestrian (%)			Cyclist (%)		
	Easy	Mod	Hard	Easy	Mod	Hard	Easy	Mod	Hard
Basaeline	93.59	87.92	85.11	73.49	65.71	57.61	80.31	73.79	61.82
+ P	93.74	88.12	85.73	73.66	65.95	58.37	80.92	74.58	62.75
+ M	94.37	88.82	87.05	77.92	70.53	63.36	85.28	80.29	74.53
+ P + M	94.63	89.19	88.02	78.28	71.01	64.47	85.73	81.13	75.59
+ M + L	94.85	89.33	88.59	80.15	72.82	65.35	86.79	82.25	76.08
+ P + M + L	**96.41**	**90.01**	**89.89**	**80.84**	**73.67**	**66.54**	**87.55**	**83.19**	**77.36**

## Data Availability

Data are contained within the article.
